# Cell Therapy in the Treatment of Female Stress Urinary Incontinence: Current Status and Future Proposals

**DOI:** 10.3390/life14070861

**Published:** 2024-07-10

**Authors:** Carmen González Enguita, María Garranzo García-Ibarrola, Jaime Jorge Tufet I Jaumont, Héctor Garde García, Raquel González López, Luis Miguel Quintana Franco, Gina Marcela Torres Zambrano, Mariano García-Arranz

**Affiliations:** 1 Urology Department, Hospital Universitario Fundación Jiménez Díaz, 28040 Madrid, Spain; jaime.tufet@quironsalud.es (J.J.T.I.J.); hector.garde@quironsalud.es (H.G.G.); rgonzalezl@fjd.es (R.G.L.); luis.quintana@quironsalud.es (L.M.Q.F.); 2 Sheikh Khalifa Medical City, Abu Dhabi P.O. Box 5166, United Arab Emirates; dr.torresz@gmail.com; 3 Instituto Investigaión Sanitaria Fundación Jiménez Díaz (IIS-FJD), 28040 Madrid, Spain; mariano.garcia@quironsalud.es

**Keywords:** stress urinary incontinence, stem cell, clinical trials, stem cell therapy, stem cell transplantation, regenerative medicine

## Abstract

Background: Stress urinary incontinence (SUI) is a common condition with a significant impact on the quality of life of female patients. The limitations of current treatment strategies have prompted the exploration of new effective and minimally invasive alternative approaches, including cell therapy. Methods: A literature search was conducted to update the current clinical status of stem cell therapy in the management of female stress urinary incontinence. Results: Over thirty clinical studies have been designed to assess the feasibility, safety and efficacy of cell therapy for female SUI. Despite differences in cell types and protocols, the overall treatment procedures were similar. Standard subjective and objective assessment tools, and follow-up periods ranged from 6 weeks to 6 years have been used. Cell injection has shown to be a safe therapy in the treatment of female SUI. However, the results from more recent randomized trials have shown less promising results than expected in restoring continence. Heterogeneous research methodologies using different cell types and doses make it difficult to draw conclusions about effectiveness. Several key points remain that need to be further explored in future clinical trials. Conclusion: To advance in the development of cell therapy, it is essential to know the mechanisms involved to be able to direct it properly, its efficacy and the durability of the injected cells. Rigorous and homogenized preclinical and clinical studies that demonstrate its scope and improve its application are necessary for validation in the treatment of female SUI.

## 1. Introduction

Stress urinary incontinence (SUI) is the most common form of urinary incontinence. It is defined as “the involuntary loss of urine when bladder pressure exceeds urethral closure pressure due to physical activity, exercise, coughing or sneezing” [1]. The incidence of urinary incontinence in the female population ranges from 10% to 40%, rising with age [2]. Several factors are associated with the risk of SUI, such as childbirth, menopause, strenuous physical work, smoking, obesity and chronic cough [3].

Although SUI is not a life-threatening condition, it has adverse physical, social and psychological consequences, leading to low self-esteem, reduced quality of life and social isolation in patients. SUI is often considered a normal part of aging and patients try to cope with it at a high economic and social cost [4].

Female SUI often has a multifactorial cause with functional defects of the urinary tract support structures, the bladder sphincter and morphological nerve damage. The underlying mechanisms for the onset and progression of SUI are not completely understood, but it is well known that the integrity of the external and internal urinary sphincters is necessary to maintain the continent function. Furthermore, somatic and autonomic nerves, fascial and ligament structures and urethral mucosa play an important role in the urinary continence mechanism. Studies on the molecular mechanisms associated with the pathogenesis of SUI further suggest that the occurrence of this condition may be influenced by various neurogenic, muscle and connective tissue-derived molecules [5,6].

Conservative management of female SUI involves behavioral therapy, biofeedback and pelvic floor muscle training. These treatments are generally simple, cost-effective and have a low likelihood of side effects, without interfering with possible future treatments if necessary [5,7]. When conservative approaches fail, more invasive procedures such as midurethral sling implantation, injection of a bulking agent and Burch colposuspension may be recommended. Surgical intervention provides good treatment against SUI but has a risk of complications [8]. The mid-urethral sling has the benefit of requiring less intervention time. However, several organizations have frequently issued warnings against the use of mesh materials in the treatment of female urinary incontinence due to the result of many severe adverse events [9]. The implantation of the artificial urinary sphincter, recommended for women with moderate to severe SUI, has been shown in systematic reviews to provide excellent functional outcomes, with complete continence rates ranging from 61.1% to 100%. However, these benefits come with high morbidity, with significant explantation and erosion rates [10].

In this context, new and less invasive alternatives, such as functional magnetic stimulation aimed at improving the contractility of pelvic floor muscles and the use of platelet-rich plasma or stem cell transplantation focused on enhancing tissue elasticity and regeneration, are being investigated [11].

Targeting the regeneration of the damaged sphincter, improving the function of external (striated) and internal (smooth) muscles, as well as sphincter blood circulation, cell therapy offers a promising approach for the treatment of SUI [6,12,13]. Different types of multipotent cells from different tissues are being investigated. Some study results have shown that cell therapy is feasible and safe [6,14,15]. However, the best use of cell technology is still under investigation, and several key issues need to be addressed, such as the optimal cell type and dose used to regenerate the sphincter, the development of surgical guidance to facilitate precise cell application and adequate tools to monitor muscle regeneration during the follow up [16,17]. It should be noted that these novel approaches are out of current guidelines and should be proposed only in referral centers for functional urology within the context of official clinical trials.

The aim of this review is to provide an update to the current clinical trials designed for the clinical development of cell therapy to treat female SUI. It summarizes the types of cells and doses, an injection route tested in the different protocols is established, their challenges are outlined and a research roadmap for the future is proposed.

## 2. Cell Therapy in Stress Urinary Incontinence

**Figure 1:**
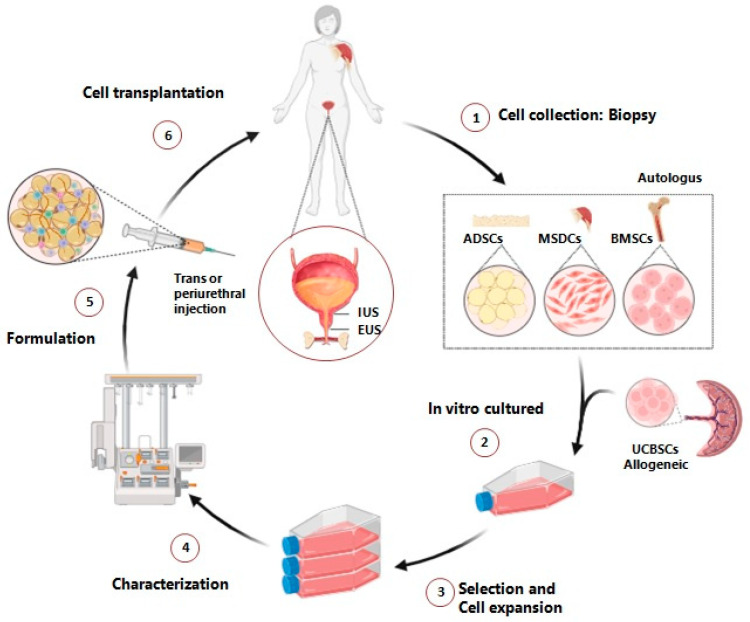
Stem cell injection therapy in female SUI.

Advances in understanding the pathology and underlying molecular mechanisms of urinary incontinence have allowed for the exploration of the potential of stem cells to restore continence [6]. Due to ethical considerations and potential complications associated with the use of embryonic stem cells, research on SUI has mainly focused on adult stem cells. The isolation and transplantation of these cells (Figure 1) present in tissues such as skeletal muscle, adipose, bone marrow, blood, urine and dental pulp have shown promising functional results in the restoration of urinary sphincter defects [12,18,19]. Preclinical research, initially aimed at demonstrating the differentiation capacity of stem cells into various cell lineages such as smooth tissue cells [20], has provided insight into the underlying molecular mechanisms involved in MSC-mediated treatment of SUI [21] and has demonstrated the potential of stem cells to safely treat SUI, both in monotherapy and in combination with its secretomes, in terms of muscle repair and regeneration, nerve regeneration and vascular preservation [6,14,21].

### 2.1. Skeletal Muscle-Derived Cells for SUI

Skeletal and smooth muscles have been recognized as an essential source of progenitor or satellite cells responsible for muscle regeneration. In addition to skeletal muscle-derived cells (SkMDCs), muscle has also been identified as a valuable source of stem cells, other than satellite cells, which possess the ability to differentiate into other cell lineages called muscle-derived stem cells (MDSCs) [22].

MDSCs are a heterogeneous population of multipotent cells with different phenotypes depending on the stage of differentiation, and are the precursors of various connective tissue cells, such as myocytes and satellite cells. From these cells, mesenchymal, neuronal and endothelial lines can be also derived, which are highly beneficial for sphincter regeneration and the neural functions involved in continence [6,18,23,24]. Experimental studies with rodent, canine and non-human primate SUI models have demonstrated their effectiveness in the regeneration of the damaged urinary sphincter and have shown evidence of the formation of myotubes, neoangiogenesis and nerve regeneration after their periurethral injection [25]. These cells have the capacity to induce both structural and functional regeneration, as evidenced by significant improvements in the intravesical closure pressure of the urethra and a greater contractile function of the urethral sphincter [26,27].

MDSCs can be obtained with muscle biopsy under local anesthesia, resulting in low morbidity. However, isolated autologous MDSCs must first be expanded in vitro and need to be cultured for a certain time before injection into the urethral sphincter [25,28]. Unlike other types of cells, the advantage of MDSCs is that cells injected into the urethral sphincter contain myotubes and myofibers, which are essential for sphincter function and can also work as a blocking agent [13,15].

### 2.2. Mesenchymal Stem Cell for SUI

MSCs can be isolated from a variety of tissues, including bone marrow, adipose tissue, umbilical cord, endometrial and oral mucosa. In addition to their regenerative ability, MSCs also have anti-inflammatory and immunomodulatory characteristics. An important advantage of MSCs is the possibility to use them allogenically, which allows for the selection of MSCs with a higher potential from donors [29].

#### 2.2.1. Adipose-Derived Stem Cells for SUI

ADSCs (adipose-derived stem cells) are one of the most commonly used types of stem cells for autologous and allogenic transplants thanks to their abundance and availability from adipose tissue. ADSCs are multipotent stromal cells and can differentiate into adipogenic, chondrogenic, myogenic and osteogenic cells. They can differentiate into myogenic cells in the presence of induction factors after periurethral injection, assuming a smooth muscle phenotype at around 8 weeks. Unlike MDSCs, ADSCs evade detection through lymphocytes and immune rejection by expressing specific and non-specific marker proteins on their surface. Furthermore, these cells show high prolonged proliferation even at low serum levels, highlighting their superiority over MDSCs [17,30].

ADSCs are particularly noteworthy for their potential to promote revascularization and neuronal and mesodermal regeneration. Preclinical studies in murine, rabbit and porcine SUI models have demonstrated that they can promote the recovery of urethral function by myogenic differentiation as well as neuroregeneration [31]. Additionally, cultured ASCs secrete various angiogenesis-related cytokines, including vascular endothelial growth factor and hepatocyte growth factor. Zhao et al. confirmed the synergy of stem cells and cytokines, detecting an increase of elastin, collagen and smooth muscle contents 4 weeks after ADSCs and nerve growth factor implantation [32]. Scherer et al. evaluated the effectiveness of ADSCs in treating detrusor underactivity in men. The study demonstrated significant improvements in maximum and mean urinary flow rates, reduced residual urine volume and increased maximum detrusor pressure and bladder contractility index in pressure-flow studies, highlighting the potential of ADSCs to restore bladder function effectively [33].

Rats treated with adipose tissue-derived stem cells (ADSC) that exhibited normal voiding patterns showed a significantly higher smooth muscle content compared to the control group and the ADSC-treated rats with voiding dysfunction. However, no significant differences were observed in collagen content between the different treatment groups or between rats with normal and abnormal voiding within the ADSC-treated group. The treatment with ADSC shows significant therapeutic potential for SUI by improving elastin and smooth muscle contents in urethral tissue, suggesting an enhancement in urethral sphincter function. These changes are due to both the direct differentiation of ADSC into cells necessary for urethral function and the secretion of trophic factors that promote host tissue regeneration [21].

#### 2.2.2. Other Mesenchymal Stem Cells for SUI

MSCs such as bone marrow stem cells (BMSCs), umbilical cord blood stem cells (UCBSCs), urine-derived stem cells (UDSCs) and amniotic fluid stem cells (AFSCs) have been assessed in vitro and in vivo to treat urinary incontinence [14,21]. Preclinical trials have shown that MSCs can enhance the repair of damaged tissue, either through direct integration and replacement of damaged tissue (differentiation) or through secreted factors that influence the recipient’s response mechanisms (paracrine effect) [34]. Despite the promising preliminary outcome, the therapeutic potential of these sources of MSCs has been tested in small animal models and only UCBSCs and TNCs have been translated to clinical trials [21,35].

UCBSCs exhibit a superior differentiation capacity compared to other adult stem cells. One advantage is their easy acquisition process, as it does not require an invasive procedure. This type of stem cell has a low risk of causing graft-versus-host diseases. Its advantages over other types of stem cells suggest that it could be a promising approach for the treatment of stress urinary incontinence [36].

Multipotent endothelial cells (EPCs) can be taken from peripheral blood to produce total nucleated cells (TNCs) and can be used for the treatment of UI. The role of growth factors and the effects of platelet-rich plasma have been investigated with in vitro and in vivo studies. This effect has been associated with several growth factors produced by platelets (platelet-derived growth factor, transforming growth factor β1, fibroblast growth factors, vascular endothelial growth factors, etc.) [28,37].

## 3. Materials and Methods

A literature search was performed aiming to review the most relevant available literature on the current status of stem cell therapy applied to the field of female SUI management. The following relevant keywords (stress urinary incontinence) AND (regenerative therapy OR cell therapy OR tissue therapy OR tissue transplantation OR stem cell transplantation OR stem cells) were used to conduct a search in PubMed, Cochrane and Embase (up to November 2023) and the search method was modified for each database. Clinical trials, meta-analysis, systematic reviews, reviews, human studies and female studies were prioritized. Simultaneously, the ongoing, future and completed clinical trials registered in *ClinicalTrials.gov* and *EU Clinical Trials Register* were identified. Two reviewers independently screened titles and abstracts of the records that were retrieved through the database searches. We also performed a manual search to include additional relevant articles.

A total of 311 results were retrieved after duplicates were excluded from database searching. Twenty-five clinical trials were identified from clinical trial databases. After removing duplicates, a total of 319 records were filtered. From the initial search, 258 were excluded during the title or abstract screening. Sixty-one records were selected for full-text reviews. Only data obtained from clinical studies involving female patients with SUI treated by cellular injection were eligible for inclusion in this study. Twenty-eight posted studies were included in this review, and five additional trials were identified.

## 4. Results

### 4.1. Clinical Trials on Cell Injection for Female Stress Urinary Incontinence

More than thirty prospective single-center or multicenter and follow-up studies have been designed to assess the feasibility, safety and efficacy of cell therapy for the treatment of female SUI. A summary of their main characteristics, type and dose of cell, transplantation routes and test outcomes are presented in Table 1, Table 2 and Table 3. Several systematic reviews have been published with their results [17,28,38,39,40,41,42,43,44].

Regarding the source of stem cells, the designed clinical trials can be classified as trials with muscle-derived cells or trials with non-muscle-derived cells.

Autologous MDSCs from different muscles have been the most widely used stem cells in the treatment of female SUI. With a follow-up of 12 weeks to 72 months, 15 prospective clinical studies [45,46,47,48,49,50,51,52,53,54,55,58,59,60,61,62,63] and two long-term follow-up studies have been published [56,57]. Among these studies, five were randomized clinical trials, including a multicenter placebo-controlled clinical trial [59] and a randomized pilot study comparing MDSC treatment versus conventional surgery [62].

In a minor number of trials, other sources of stem cells have been evaluated. A total of 73 women with SUI have participated in five clinical treatment studies with ADSCs [64,65,66], umbilical cord blood stem cells (UCBSCs) [67] and TNCs with platelet-rich plasma [68] (Table 2). And recently, 30 women with SUI have participated in the first randomized pilot trial to explore the use of mucosa-derived stem cells in the treatment of female SUI [69].

**Table 2:** Clinical trials with no muscle-derived cells for treatment of female SUI.

Publication Author/Year/Clinical Trial Identifier	Cell Type	Study Design	Females (n)	Follow-Up (m)	Cell Dose (×10^6^ Cells)	Cellular Injection		Clinical Evaluation	
						Approach Via	Number Injection	Functional Evaluation	PROMs
Kuismanen et al. 2014 [64]	ADSCs	SAS	5	12	2.5–8.5 (2.4–4 mL)	Transurethral (Cystoscope)	4	Cough test MUCP/PT	VAS/UDI-6/UISS/ IIQ-7
Arjmand et al. 2017 [65]	ADSCs	SAS Phase 1	10	6	1.18 (10 mL)	Trans and periurethral Transvaginal (urethroscope)	3	PT/Qmax	ICIQ-SF
Garcia-Arranz et al. 2020 [66] NCT01804153	ADSCs	SAS	10	12	40 (2 mL)	Transurethral (Cystoscope)	7–8	PT/UDS Cough test	SF-12 ICIQ-SF
Lee et al. 2009 [67]	UCBSCs	SAS	39	12	430 ± 190 (2 mL)	Periurethral (Cystoscope)	2	MUCP	PSQ
Shirvan et al. 2013 [68]	TNCs MNCs	SAS	9	6	TNCs: 676 ± 122 MNCs: 188 ± 32 (10 mL)	Transurethral (Cystoscope)	10	PT/UDS Cough test	ICIQ-UI ICIQ-QoL
Mahboubeh et al. 2023 [69] IR.kmu.rec.1395.343	Mucosa Derived SCs	RCT/ Surgery	30(15)	24	64 (4 mL)	Periurethral (Cystoscope)	8	Cough Test	IIQ

MUCP: Maximal Urethral Closing Pressure; PSQ: Patient Satisfaction Questionnaire; IIQ: Incontinence Impact Questionnaire ICIQ-QoL: Incontinence Modular Questionnaire-Quality of Life; ICIQ-UI: International Consultation on Incontinence Questionnaire-Urinary Incontinence; ICIQ-SF: International Consultation on Incontinence Questionnaire Short Form; MNCs: Mononuclear cells; SF-12: Short-Form Health Survey; UISS, Urinary Inventory Stress test.

This update also includes nine additional trials, mainly with muscle tissue-derived cells, registered on the Clinical Trials Government website and the EU Clinical Trials Register. There were three completed trials with no posted results, two ongoing studies and one that is not yet recruiting. In addition to two multicenter randomized placebo-controlled clinical trials [70,71,72] and a follow-up dose study of a cohort of patients participating in the SUITE trial [73], a phase 3 clinical study was designed to find the optimal cell count for functional regeneration of urethral sphincter deficiency in female patients with predominantly intrinsic sphincter deficiency of moderate severity. The results of these multicenter randomized clinical trials, the largest ever conducted, have recently been published on the official clinical trials registry websites [70,71,72], although the final analysis has not yet been published (Table 3).

**Table 3:** Additional clinical trials registered with stem cells for treatment of female SUI.

Clinical Trial Identifier	Cell Type	Study Design	Females (n)	Follow-Up (Months)	Cell Dose (×10^6^ Cells)	Cellular Injection Route	Clinical Evaluation		STATUS
							Functional Test	PROMs	
SUITE Study Eudra CT: 2009–011797-15	MDSCs	RCT/ Placebo Phase 2	242	8	LD: 0.2 HD:10	Transurethral	VD/PT IEF score	IQoL/VAS CGI/PGI	Completed 2011
Innovation Study Eudra CT: 2010–021871-10	MDSCs	RCT Phase 3	377(252)	12	0.2 (2 mL)	Transurethral (ultrasound)	VD IEF score	I-QoL	Completed 2023 [71]
Eudra CT: 2014–001656-34	MDSCs	Phase 2 FUS	42	48	LD: 0.2 HD:10	Transurethral	VD/IEF score/PT	I-QoL/VAS CGI/PGI	Completed 2021 [73]
NCT01893138 Eudra CT: 2014–002919-41	MDSCs	RCT/ Placebo	297(199)	24	150 (4 mL)	Transurethral	VD/PT IEF score	IQoL	Completed 2021 [70,72,74]
NCT02334878	BMSCs	NRCS Phase 3	50(25)	12	20–30	Periurethral	Cough test/UDS	IQoL	Completed 2016
NCT04446884	ADSCs/ Collagene	SAS Phase 1	10	12	6 (3 mL)	Paraurethral	NR	NR	Completed 2019
NCT03104517	MDSCs	RCT Phase 3	96	12	150	Transurethral	VD IEF score	IQoL	Recruiting
NCT05534269	MPCs	RCT DRS Phase 2	70	6	NR	Transurethral	VD IEF score PT/MRI	ICIQ-UI SF LUTS-Qol VAS/PGI-I	Recruiting
NCT03997318	MDSCs	RCT	24	12	150	Transurethral	VD IEF score	IQoL	Not yet recruiting

NRCS = Non-randomized control study; LUTS-Qol: Lower Urinary Tract Symptoms quality of Life.

### 4.2. Stem Cell Injection Procedure

Despite differences in cell types and study protocols (Table 1, Table 2 and Table 3), treatment procedures were generally similar among the included studies. The process begins with the removal and isolation of desired cells from the patient’s tissue, followed by their culture and expansion in vitro until a certain number of cells is reached. A second minimally invasive intervention is then performed to inject the formulated cell product into the urinary sphincter or specific surrounding areas (Figure 1).

Autologous MDSCs were obtained using open-muscle biopsy from different muscles and in different amounts of tissue under local anesthesia. ADSCs used in three studies [64,65,66] were collected using liposuction of subcutaneous lower abdomen fat tissue, also under local anesthesia. The mixture of TNCs/MNCs and platelets was prepared according to standard procedures from peripheral blood collected from patients one day before implantation [68]. And mucosa-derived SCs were collected from a sample of lower lip mucosal tissue under local anesthesia [69]. The only trial performed with allogeneic cells was by Lee et al. in which UCBSCs were obtained from a donor-based banking system [67].

Direct transurethral injection guided by endoscopic or ultrasound methods has been the main route of cell delivery used in most published studies, along with periurethral and transvaginal periurethral injections (Table 1 and Table 2). The most common injection site has been at the mid-portion of the urethra around the omega-shaped rhabdosphincter or submucosa [28].

### 4.3. Stem Cell Dose

There is a wide variation in the number of cells injected among the trials performed [17,39]. In female SUI treatments, the number of transplanted cells ranged from 0.2 to 200 × 10^6^ cells for MDSC and from 2.5 to 40 × 10^6^ cells for ADSC therapy. The total injection volume was <10 mL in most trials, delivered in a number of injections that ranged from 1 to 26 (Table 1 and Table 2).

Two randomized dose-ranging feasibility studies [48,52] and two phase 1/2 studies with identical selection criteria and outcome measures (NCT00847535/NCT01008943) were conducted to determine the optimal dose of stem cells to implant into the urethral sphincter [54]. The pooled analysis of their results showed that injection of autologous muscle tissue-derived cells at cell doses of 10, 50, 100 and 200 × 10^6^ cells is safe and that a major response is observed at the higher doses of 100 and 200 × 10^6^ cells [54,75].

Three multicenter, randomized, placebo-controlled clinical trials have been conducted to evaluate the effect of implanting one or two treatments of 150 million autologous MSCs into the urethral sphincter for the treatment of female SUI [59,70,71,72]. The first study (NCT01382602) was conducted at 10 research centers in Canada, the UK and Germany (Table 1). A total of 143 women, out of 246 planned, were randomized 2:1 to receive 150 million autologous muscle-derived cells for urinary sphincter repair (AMDC-USR) or placebo. Their results indicated that injecting these cells was safe and well tolerated, although an interim analysis showed a higher-than-expected placebo response rate, leading to a halt in recruitment [59]. The second study, a phase 3 study (NCT01893138), included 297 women with SUI [70,72,74]. As in the previous trial, the patients were randomized 2:1 to receive 150 × 10^6^ AMDC-USR or the placebo. After 12 months, participants were unblinded, and those in the placebo group could receive treatment with AMDC-USR. Participants were followed for 2 years after initial treatment. SUI was monitored using a 3-day stress incontinence episode frequency diary (SIEF) and quality of life questionnaires (Table 3).

The results showed that treatment with AMDC-USR was safe and durable for up to 2 years, with variable therapeutic effects. The reduction in SIEF frequency correlated with improvements in all quality-of-life scores at 12 months. In addition, treatment-related serious adverse reactions were rare (<1%) and no safety signals associated with AMDC-USR were detected [70,72]. A double-blind, two-stage trial is underway to finalize data for regulatory approval and commercialization of AMDC-USR (NCT03104517) (Table 3) [75]. Parallel to these studies, a third multicenter clinical trial with a lower dose (0.2 × 10^6^ cells/2 mL) of skeletal-muscle-derived cells was conducted. The Innovation Study (Eu Clinical Trials Register 2010-021871-10) enrolled 476 women with SUI across six European countries. A total of 377 patients were randomized to receive MDSCs or the placebo (2:1 ratio). The endpoints of the study were the pre–post difference in the parameters and values recorded from a 7-day voiding diary and I-QoL questionnaire. Although the final analysis has not yet been published, preliminary results available on the EU Clinical Trials Register page indicate that MDSC injection was safe and well tolerated. However, the efficacy was not as promising, as no statistically significant differences were found between the injection group and the control group, possibly due to the low dose of injected cells [71].

### 4.4. Outcomes Tests

Several subjective and objective tools to assess the results of cell therapy in the treatment of female SUI (Table 1 and Table 2) have been used. Among the subjective ones, more than ten different types of validated questionnaires (14) have been used, with the Incontinence Quality of Life Questionnaire (I-QOL) being the most commonly employed. In relation to objective tests, the cough stress test, the pad test (1H and 24H), 3- and 7-day voiding diaries and urodynamic studies have been the most widely used.

More recent clinical trials have included in their objective assessment tools the scoring of patient-reported stress incontinence episode frequency (IEF) in voiding diaries [59,70,76,77,78]. The change in IEF score has become the primary efficacy endpoint of these studies to assess the therapeutic effect of cell therapy, and the correlation between improvement scores in quality of life and reduction of incontinence symptoms, defined in terms of decreased incontinence episodes, has been proposed [79]. However, there is still no consensus on the definition of response, which remains very diverse among the trials conducted.

Additional tests have been in the works, such as functional MRI, which has recently been proposed as a useful tool for tracking injected cells and assessing urethral sphincter function at the same time [63].

## 5. Discussion

Considering the poor results obtained using synthetic bulking agents for SUI, regenerative medicine offers a promising alternative therapeutic strategy for SUI [15]. Stem cell therapies have become attractive tools due to their biocompatibility and lack of adverse inflammatory reactions [62]. If proven safe and effective, regenerative medicine could offer a minimally invasive alternative to current treatments, allowing patients to undergo a quick biopsy and receive a simple injection, thus avoiding hospitalization, extended recovery, or significant side effects associated with common current treatments.

Preclinical studies have provided information on how externally injected cells can restore continence function [14,21]. The ability of these therapies to enhance the regeneration of the damaged rhabdosphincter and improve both external and internal sphincter function has been demonstrated in experimental research [6,12]. However, doubts remain about the suitability of experimental pathology models for translating their results into clinical practice. Differences in the initial state of tissues and hormonal influences between experimental models and female patients have an impact on modeling and need to be assessed. Further studies, choosing a suitable preclinical model, are needed to avoid conflicting results [21].

### 5.1. Stem Cell Therapy Clinical Research

Interventional (single arm) phase 1/2 and pilot studies with very heterogeneous protocols have been designed initially to evaluate the viability, tolerability and effect of different types of cell injection. The first reported an approach to SUI treatment involving the implantation of myoblasts in the urethral rhabdosphincter and fibroblasts mixed with collagen in the urethral submucosa. This strategy resulted in a cure rate of 65–90% in 12 months, although it raised several questions about the likely thickening effect that could contribute to this improvement [45]. In 2008, Carr and colleagues injected autologous, myocyte-derived stem cells into the urethral sphincters of eight women with SUI, showing improvement in six patients (62.5%) at 12 months [47]. In 2013, the trial was expanded to 38 patients and different stem cell dosages, observing greater improvement with high doses (≥32 × 10^6^ cells). Since then, multiple trials with different protocols have been carried out using heterogeneous cohorts, with wide variation isolation techniques, dosing and measured efficacy parameters. Two meta-analyses have been performed to analyze the efficacy and safety of cell therapy in the treatment of SUI [17,43].

The first, by Huang et al. [43], included 890 patients (572 women) from 23 published studies. The cure rate and effective rate (cure rate plus improvement rate) showed a considerable difference between the five subgroups into which the studies were divided according to the cell type analyzed. The pooled effective rates were 92% for the subgroup of myoblasts with fibroblasts (two studies of 137 patients), 97% for the subgroup of TNCs with platelet-rich plasma (one study of nine patients), 72% for the subgroup of UCBSCs (one study, 36 patients) and 60% for the subgroup of ADSCs (one study of five patients). For the myoblast subgroup (344 patients in 12 studies), the pooled cure rate was 25% [43]. In the second, published by Mariotti et al. [17], a total of 424 patients (406 women) with SUI treated in 12 clinical studies with autologous MDSCs (nine studies) and ADSCs (three studies) were included. The analysis found a mean 41% rate of continence recovery across studies with no significant differences using MDSC or ADSC [17]. However, these results should be interpreted with caution owing to the limited number of studies and patients involved for each type of stem cell included.

On the other hand, dose-finding studies demonstrated the safety of injection of MDSCs at all doses tested (0.2–300 × 10^6^ cells) or MSCs (2–600 × 10^6^ cells) and suggested greater efficacy at higher doses [52,54]. The pooled analysis of data from two trials using autologous MDSC injections in the dose range of 10–200 × 10^6^ showed subjective improvements in all treatment groups, with the higher dose cohorts showing a higher proportion of patients experiencing at least a 50% reduction in stress leaks and pad weight [54]. At lower doses (0.2 and 10 × 10^6^ cells), the equivalence of both cell doses with regard to efficacy and safety was confirmed [80]. However, following the results of the multicenter, randomized, placebo-controlled clinical trials conducted to evaluate the efficacy of these injections, the most appropriate dose and schedule have not yet been determined [59,70,71,72]. This justifies the need for further studies to determine the optimal number of cells required for transplantation.

All studies were conducted as monotherapy except in three of them, where patients received neuromuscular electromagnetic stimulation (NMES) post-cell injection. Blaganje et al. and Schmidt’s studies highlight the potential of combining stem cell therapy with NMES; however, owing to the limited number of patients, further research is needed to elucidate NMES’s adjuvant role in cell therapy for SUI [49,61,63].

Two new pilot studies have compared the efficacy of cell-based therapy versus conventional surgical treatment. The first one was carried out by Daneshpajooh et al. [62]. Thirty female patients with SUI were randomized 1:1 in a prospective interventional study [62] to analyze the therapeutic effect of periurethral injection of MDSCs versus mid-urethral sling surgery. At one year post-treatment, ten patients (66.6%) in the MDSC group showed improvement in SUI, including five patients (33.3%) who had complete recovery of urinary continence. Improvement in incontinence was observed in 13 patients (93.3%) in the TVT control group, with complete recovery in 12 patients (%) [62]. The second was recently published by Mahboubeh et al., in which 30 women with pure SUI were assigned 1:1 to two groups: mini-sling insertion or periurethral injection of autologous mucosal stem cells with follow-up at 6 and 24 months. The results were compared using the standard Incontinence Impact Questionnaire (IIQ) for patient satisfaction as well as the Marshal test as primary objective outcomes. A negative Marshal’s test was observed in 73% of the stem cell group versus 80% in the mini-sling group (*p* = 0.6) [69].

Most trials have used a combination of subjective, patient-reported outcomes (PROMs) and objective measures to assess their main objectives, including safety and treatment efficacy. However, the definition of success varies widely between trials. Outcomes were mainly reported in terms of continence (pad-free status), recovery or improvement, whereas a quantitative (pad weight test) or qualitative (standardized questionnaire) evaluation of urinary leakage improvement was mainly unavailable. All of the studies reported some degree of improvement in SUI in the patients treated showing the potential role of SCT in the recovery of urethral sphincter function [28]. To improve the assessment of treatment success in future clinical trials, more rigorous and clinically relevant efficacy endpoints should be considered.

Patient selection is crucial in stress urinary incontinence (SUI) treatment. Age, obesity, and parity are significant risk factors that may impact treatment efficacy. Studies have shown that tailoring treatment to individual risk factors and combining conservative approaches can improve outcomes [15,38]. Future research should focus on treatment safety and efficacy in different patient profiles and SUI subtypes to optimize regenerative medicine outcomes.

Clinical trials on cell therapy for female SUI have demonstrated feasibility and safety, highlighting its significant potential as an innovative therapeutic approach. However, new randomized clinical trials have yielded less promising results, raising questions and controversy [15,17,44,81]. The lack of uniformity among studies in cell type and number, imprecise patient population definitions and varying criteria for defining efficacy prevent solid conclusions on true efficacy [17,42]. More clinical trial data are needed in order to determine whether this treatment is effective and superior to existing options or not, considering their high economic costs [82].

### 5.2. Safety Reports of Stem Cell Injection Therapy in Female SUI

Safety data from various analyses conducted to assess the effect of regenerative therapy have shown that the treatment of female SUI with stem cells, regardless of their origin, has a generally favorable safety profile [17,42,43]. Adverse events (AEs) and complications related to cell biopsies such as vasovagal syncope, dizziness, mild pain or tenderness, superficial infection, postoperative bleeding requiring stitches, wound hematoma and joint swelling were reported in a few trials [50,52,54,59]. With regard to injection and administration of the investigational cellular therapy, although some well-controlled minor complications were observed, most studies did not report serious adverse events. Some studies reported mild or spontaneously resolved AEs, such as injection site pain, dysuria, polyuria, urgency, transient haematuria, vulvovaginal pruritus and urinary tract infection, among others [55,63].

Injection site complications, such as pseudo-abscess formation and urethral erosion, have been reported, along with serious complications due to particle migration, including pulmonary embolism, which in one study resulted in a death. The use of autologous fat injections was associated with a high rate of complications, including fat embolism. Additionally, the rates of new urinary symptoms like urgency incontinence were notably higher with some agents [15,83].

Results from new studies continue to confirm the safety of cell injection. No unexpected reactions or adverse events were reported after the biopsy and transurethral cell injection. No relevant complications or serious adverse events were documented during follow-up [62,69,84]. Mahboubeh et al. compared the outcomes of stem cell therapy with mini-sling insertion in women with SUI and showed that injection of autologous adult mucosa-derived stem cells can be a safe and feasible treatment for women with SUI. Shorter procedure times and hospital stays and fewer complications were reported for the patients in the stem cell group. Dysuria and urinary tract infection were the most common complications that occurred in the stem cell group, while in the mini-sling group, additional obstructive symptoms and dyspareunia were reported [69].

Overall, stem cell therapy for SUI can be considered a promising therapy with a low risk of significant morbidity. However, it is important to be aware of the potential adverse effects and complications associated with the procedure.

### 5.3. Perspectives and Challenges in Clinical Development of Cellular Injection for Treating Female SUI

Many challenges remain, considering that several aspects of the overall efficacy of adult stem cells in the treatment of female SUI are yet to be investigated [25,34]. These include the loss of cell function after ex vivo expansion, poor engraftment or survival in vivo and the tendency of stem cells to cluster at the injection site. Moreover, understanding the mechanisms of action underlying the therapeutic effects of MSCs in vivo, the “needle effect” during stem cell injection and the paracrine effects of the cell culture secretome need to be investigated in upcoming preclinical trials designed to better understand how they may influence the final outcome of cell therapy [61].

The search for the most optimal type of stem cells for urethral sphincter regeneration awaits resolution due to the lack of conclusive evidence. The differentiation potential and regenerative capacity of stem cells play a crucial role in their suitability for treating SUI but few experiments have attempted to compare their efficacy [85]. Balancing therapeutic effects and side effects remains a pressing issue, where the use of stem cells in combined therapy may offer a future option [21]. The source and accessibility of stem cells significantly impact their clinical use. Adult stem cells can be derived from various sources, such as bone marrow, amniotic fluid, adipose or muscle tissue, umbilical cord blood, urine, tissues and even dental pulp [86]. Invasiveness of procurement procedures and safety profiles, including immune rejection, oncogenic potential and ethical considerations, are also important factors in selecting cells for clinical use. The processing and preparation of stem cells also influence their selection, as some types require complex and time-consuming procedures for collection and preparation, which ends up affecting their clinical feasibility [44].

Furthermore, issues associated with cell suspension delivery and distribution, such as is the case with the speed and method of cell suspension introduction or the insufficient injection accuracy into the external urethral sphincter, which still have to be assessed in future studies. Endoscopically or ultrasound-guided transurethral and/or periurethral injections have commonly been used for administering viable cells in female SUI treatment, although few studies have compared their results and there is no clear evidence for the superiority of one technique over the other [15,83]. Recently, a new needle-free injection method has been proposed, which could reduce cell loss due to misplacement and could be a promising option for cell transplantation [87]. Non-invasive tests for monitoring the long-term viability and functionality of implanted cells, such as imaging tests and radiotracers, could be useful but require further development before incorporation into trial protocols [84]. Nevertheless, there is still a long way to go before these can be incorporated into future trial protocols, regardless of the additional costs involved.

Cell therapies based on living medicine face challenges due to the short half-life, stability and fragility of the product. Good handling and optimization of the therapeutic potential of the cells to be injected is crucial. Controlling needle diameter to avoid breaking cells, delicate handling of the material and ensuring sterility are key aspects of treatment success. Pre-release studies of the cell product at production centers are necessary to homogenize results.

Finally, the cost of cellular products must be lowered. New approaches using allogeneic MSCs [88], stem cell culture-derived microvesicles or the targeted secretome in stem cell culture could decrease costs. Although the results of preclinical studies have demonstrated its potential and feasibility, future clinical trials should confirm its efficacy [6].

### 5.4. Future Proposals

Based on this review, we believe that allogeneic MSCs should be used in future studies. ADSCs appear to perform as the best type of all that have been assessed for urologic uses [89], and the scalability and economic benefits of allogeneic MSC make them a compelling option for the development of novel and reliable treatments [88]. Its use, on the one hand, would reduce the number of surgical interventions and the production costs of the cellular product, and on the other hand, it would shorten treatment times and optimize the potential of the cell suspension with testing on the donor and final cell product. Currently, there is no consensus on the most appropriate dose to inject, but based on the dose studies carried out and on our previous experience with this type of stem cells, we believe that a homogenous dose of 40 million cells would be adequate and effective [66]. We propose direct transurethral injection guided by an endoscopic method as the best treatment route, and, according to most clinical trials, a second treatment and longer follow-ups would be recommended. Another future option that would reduce costs considerably could be the use of the secretome generated in MSC cultures, but its efficacy has not yet been demonstrated.

The results of the evaluation systems, as well as the selection of the study population, are other aspects to take into account when designing new clinical trials. According to the clinical evidence, not all patients may be suitable for these therapies. Women with great urethral hypermobility would not be the most recommended candidates to include [38,75]. In relation to the evaluations to be carried out, we consider that pad tests and urodynamic studies are suitable tools for a correct analysis of the results, as well as a 3-day-voiding diary to assess the reduction of IEF score, together with a quality of life questionnaire such as the IQoL. Additionally, it is important to note that pad tests and urodynamic studies have been utilized in few studies. Therefore, their promotion in future studies is essential to evaluate treatment success comprehensively. Finally, training in the use and manipulation of cell therapy products is necessary when large trials are carried out to ensure the viability of the cell treatment and, therefore, its therapeutic effect.

## 6. Conclusions

Despite the great progress that has been made in the field of cell therapy for the treatment of female SUI, the demonstrated safety and the promising initial efficacy data generated by clinical studies, there are still several aspects to be investigated and improved. The inherent limitations of cell therapy and heterogeneity in clinical trials make it difficult to establish clear protocols, justifying the need for further, more homogeneous clinical studies. There is still a need for consensus on the objective assessment test and target population in future clinical trials, as well as the necessity to improve the handling of advanced therapy drugs (live drugs), and to reduce the cost of the treatments in order to include them as a minimally invasive option in the future.

## Figures and Tables

**Table 1:** Clinical trials with muscle-derived cells for treatment of female SUI.

Publication Author/Year/Clinical Trial Identifier	Cell Type	Study Design	Females (n)	Follow-up (m)	Cell Dose (×10^6^ Cells)	Cellular Injection		Clinical Evaluation	
						Approach via (Guided)	Number Injection	Functional Evaluation	PROMs
Mitterberger et al. 2007 [45]	M,F	PS	123	12	M: 5.1–36 F: 5.4–60	Transurethral (ultrasound)	15–18	UDS	IQoL
Mitterberger et al. 2008 [46]	M,F	PS	20	24	M: 10–30 F: 14–60.6	Transurethral (ultrasound)	15–18	UDS	IQoL
Carr et al. 2008 [47]	MDSCs	PS	8	12	18–22	Trans and peri- urethral (Cystoscope)	2–4	VD PT	NR
Sèbe et al. 2011 [48]	MDSCs	RCT	12	12	10/25/50	Transurethral (endoscope)	2	VD PT	Contilife Q
Blaganje et al. 2012 [49,50] NCT01355133	MDSCs	PS	38	6	1–50	Transurethral (ultrasound)	26	Stress test VD/PT	PGI-I IQoL/VAS
Surcel et al. 2012 [51]	MDSCs	PCS	82(4)	12	NR	Periurethral (ultrasound)	20	UDS	IQoL
Carr et al. 2013 [52]	MDSCs	RCT	38	18	LD: 1–28 HD: 36–128	Trans and peri- Urethral (Cystoscope)	8	PT	UDI-6 IIQ-7
Peters et al. 2014 [53,54]	MDSCs	PS * Phase 1/2	80	12	10–200 (4 mL)	Trans and peri- urethral (Cystoscope)	8	3d-VD PT	UDI-6 IIQ-7
Stangel-Wojcikiewicz et al. 2014 [55]	MDSCs	PS	16	24	0.6–25	Transurethral (endoscope)	3	Cough tests UDS	Guadene-Q
Cornu et al. 2014 [56]	MDSCs	FUS	11	72	10/25/50	Transurethral (endoscope)	2	PT	PGI-I USP
Stangel-Wojcikiewicz et al. 2016 [57]	MDSCs	FUS	16	48	0.6–25	Transurethral (endoscope)	3	Not evaluated	IQoL
Sharifiaghdas et al 2016 [58] NCT01963455	MDSCs	SAS Phase 1	10	36	38.6	Transurethral (endoscope)	4	Cough tests 1h-PT,UDS	IIQ-7
Jankowski et al. 2018 [59] NCT01382602	MDSCs	RCT/ Placebo Phase 3	143(93)	12	150 (4 mL)	Transurethral (Cystoscope)	9	PT/VD (IEF score)	UDI-6/IIQ-7 IQoL/ISI GQoL
Sharifiaghdas et al. 2019 [60] NCT02156934	MDSCs	SAS Phase 2	20	24	≥50 (10 mL)	Transurethral	NR	Cough tests 1h-PT,UDS	IIQ-7, UDI-6
Blaganje & Lukanovic et al. 2022 [61]	MDSCs	SAS Phase1/2	38	24	0.2 (2 mL)	Transurethral (ultrasound)	26	UDS/PT VD (IEF score)	IQoL CGI-S VAS
Daneshpajooh et al. 2022 [62] IR.kmu.rec.1395.349	MDSCs,F	RCT/ Surgery	30(15)	12	30	Periurethral	2	Cough test PT	ICIQ-UI-SF IQoL
Schmidt et al. 2023 [63]	MPCs	RCT Phase 1	10	6	80–100	Transurethral (ultrasound)	NR	PT/MRI UDS	ICIQ-UI-SF

* Pooled data from two phase 1/2 clinical trials: NCT00847535/NCT01008943. CGI-S: Clinical Global Impressions Severity scale; FUS: Follow-up Study; GQoL: Global Quality of Life Assessment; ICIQ-UI-SF: International Consultation on Incontinence Questionnaire–Urinary Incontinence short form; HD: High Dose; IEF: Incontinence Episode Frequency; IIQ-7: Incontinence Impact Questionnaire short form; IQoL: Incontinence quality of life questionnaire; ISI: Index for Urinary Incontinence; LD: Low Dose; M,F: Myoblast, Fibroblast; MPCs: Muscle Progenitor Cells; MRI: Magnetic Resonance Imaging; NR: Not reported; PCS: Prospective Cohort Study; PGI-I: Patient Global Impression Improvement; PS: Prospective Study; PT: Pad Test; Qmax: Maximum urinary flow; RCT: Randomized Clinical Trial; SAS: Single-Arm Study; UDI-6: Urogenital distress inventory-short form; UDS: Urodynamic Study; USP: Urinary Symptom Profile; VAS: Visual Analogical Scale; VD: Voiding diary.

## Data Availability

No new data were created or analyzed in this study. Data sharing is not applicable to this article.
